# Methods of Building up Health

**Published:** 1883-05

**Authors:** 


### HALL'S JOURNAL OF HEALTH AND MISCELLANY.

MONTHLY.

E. H. GIBBS, A. M., M. D., EDITOR.

FOUNDED IN 1854.

### METHODS OF BUILDING UP HEALTH.

Careful observations of medical men in all civilized countries prove that the highest mental and physical powers are developed by those methods that minister to our creature comforts, and lift the weight of care.

The average term of life of the nobility of England is sixty years. They have comfortable homes, enjoy greater freedom from care and are better nourished than any other class of men in the world, and they are active in the pursuit of pleasures that conduce to health. Next comes a class of literary men whose means afford them opportunities to pursue their studies and live comfortably, independent of the anxieties of business. This class attains an average life of fifty years. And thus we look down the scale until at the bottom we find the poor and miserable creatures, victims of starvation, intemperance and disease, whose average term of life is less than twenty years.

It is evident, then, that the reasonable expectation of life of any individual depends more upon his habits, his freedom from care, and on proper nourishment, than upon the fact that he may have inherited a vigorous constitution. We often find that such children become feeble and short lived men and women, while the most unpromising children grow up strong and healthy, with proper care.

It is unfortunate that thousands of parents refuse to profit by the knowledge and experience of competent medical men in regard to the care of their children, and cling to the most absurd theories about diet and other matters of vital importance.

We often read the story more or less true of the early life of some man who has become eminent; how he lived in a log house, and ov e a horse on the tow path, or split rails, and acquired much learning by the dim and uncertain gleam of a pine knot; but we are not told that he did not have enough to eat or enough sleep, for if we were, we should discredit the whole story; since no child who is habitually under-fed and under-slept, will ever become eminent. Starvelings may become "Dudes" but never brilliant men, though born in a palace.

The essentials to sound health of mind and body are freedom from care and anxiety.

An abundance of wholesome and well-prepared food.

Eight hours sleep out of every twenty-four hours.

All the labor or exercise daily that can be taken without great fatigue.

A constant supply of pure, fresh air for the lungs.

Scrupulous cleanliness.

Clothing adapted to the changes of the seasons.

An honorable and remunerative occupation.

The confidence and respect of your fellow men.

A variety of foods is best.

Learn to eat with a relish whatever is wholesome; but avoid rich pastry, particularly *pies.*

The best exercise is horseback riding. It will contribute more than any thing else to the cure of almost every curable disease; and particularly disease of the lungs. I have for years recommended this treatment as the only one that offered any hope in advanced cases of lung disease, and the best results have frequently followed this plan when properly carried out.

But the great trouble is a want of courage to surmount obstacles. A patient may be in that condition where a prompt compliance with this advice will almost surely restore him to health, or where hesita tion and delay will soon place him beyond hope. Unfortunately three patients in four will do nothing, because that is easiest. Where there is great debility there is but little ambition to overcome it; just as a person under the influence of opium dislikes to be aroused from his slumber; but in either case there is safety only in action prompt, vigorous and long continued.

